# Novel Therapies in Glioblastoma

**DOI:** 10.1155/2012/428565

**Published:** 2012-02-28

**Authors:** James Perry, Masahiko Okamoto, Michael Guiou, Katsuyuki Shirai, Allison Errett, Arnab Chakravarti

**Affiliations:** Department of Radiation Oncology, Arthur G. James Comprehensive Cancer Center and Richard L. Solove Research Institute, The Ohio State University, Columbus, OH 43210, USA

## Abstract

Conventional treatment of glioblastoma has advanced only incrementally in the last 30 years and still yields poor outcomes. The current strategy of surgery, radiation, and chemotherapy has increased median survival to approximately 15 months. With the advent of molecular biology and consequent improved understanding of basic tumor biology, targeted therapies have become cornerstones for cancer treatment. Many pathways (RTKs, PI3K/AKT/mTOR, angiogenesis, etc.) have been identified in GBM as playing major roles in tumorigenesis, treatment resistance, or natural history of disease. Despite the growing understanding of the complex networks regulating GBM tumors, many targeted therapies have fallen short of expectations. In this paper, we will discuss novel therapies and the successes and failures that have occurred. One clear message is that monotherapies yield minor results, likely due to functionally redundant pathways. A better understanding of underlying tumor biology may yield insights into optimal targeting strategies which could improve the overall therapeutic ratio of conventional treatments.

## 1. Introduction

Glioblastoma (GBM) is a grade IV glioma and accounts for approximately 75% of all high-grade gliomas with approximately 9,000 new cases per year diagnosed in the United States alone, making it the most common adult brain tumor. GBM is the most aggressive glial neoplasm, and despite advances in medical management, the outcomes remain quite poor. The current standard of care for high-grade glioma patients is maximum surgical resection combined with radiation and concomitant and adjuvant temozolomide (TMZ) therapy. The addition of radiotherapy for the treatment of GBMs led to the first significant improvement in patient survival starting in the late 1970s. More recently, Stupp et al. have shown that the addition of the chemotherapeutic agent TMZ can increase survival further to approximately 15 months.

## 2. Development of Standard of Care (RT + TMZ)

Surgery is a critical component of standard of care, allowing histological diagnosis, but more critically, tumor debulking. This greatly reduces the number of cells to be killed by radiation or chemotherapy. It also decreases intracranial pressure which, depending on the location of the tumor, may result in recovery of CNS function or decrease in usage of corticosteroid. Recently, the effectiveness of aggressive surgical resection on survival was suggested by some prospective analyses [1–3].

Unfortunately, most glioblastomas recur following surgery. The efficacy of radiation therapy (RT) was reported in the 1970s [4, 5], and RT has become a standard adjuvant therapy in patients with malignant glioma. In 2005, the efficiency of concomitant and adjuvant TMZ was suggested by a phase III study that was conducted by the European Organization for Research and Treatment of Cancer (EORTC) and the National Cancer Institute of Canada (NCIC) [6]. In the EORTC/NCIC study, a total of 573 patients with newly diagnosed glioblastoma were enrolled. The authors reported the combined therapy of TMZ and RT increased median survival time (MST) when compared with RT-alone (14.6 months versus 12.1 months, *P* < .001). At the 5-year analysis of this study, the 5-year overall survival rate was 9.8% for the combination therapy group versus 1.9% for the RT alone group (*P* < .001), with a median follow-up of 61 months [7]. With this strong evidence, combination therapy with TMZ and RT is widely prescribed and currently considered the standard treatment for patients with newly diagnosed glioblastoma.

Some investigators had suggested that the epigenetic silencing of a DNA repair enzyme named *O*-6-methylguanine-DNA methyltransferase (MGMT) by promoter methylation was associated with good prognosis for patients with glioblastoma treated with alkylating agents such as TMZ [8, 9]. In agreement with previous studies, patients with a methylated MGMT promoter had significantly improved MST when compared with patients with an unmethylated MGMT promoter (21.7 months versus 15.3 months, *P* < .001) in the EORTC/NCIC trial [10]. Furthermore, this study indicated that patients with an unmethylated MGMT promoter received less benefit from the combined therapy. MGMT promoter methylation status is widely used to predict the efficacy for combination therapy of RT and TMZ for newly diagnosed glioblastoma.

Although the combination therapy of RT and TMZ has become standard, most patients will still eventually recur. Thus the development of a new treatment strategy is needed in order to overcome the resistance of glioblastoma to current therapy. One strategy is increasing the intensity of radiation dose. However, neither radiosurgery boost [11] nor brachytherapy boost [12] shows improvement in survival. Another strategy is the optimization of TMZ usage by approaches such as dose-dense regimens. RTOG 0525/EORTC 26052-22053, a prospective randomized trial, was conducted by the Radiation Therapy Oncology Group International (RTOG) and EORTC. It aimed to determine whether a dose-dense TMZ regimen is more effective than the standard TMZ regimen in the adjuvant setting, and the results of this study showed no significant difference between the two arms [13]. Moreover, the adverse effects, especially in the field of lymphopenia and fatigue, were significantly increased in the dose-dense arm.

## 3. Technological Advancements in GBM Therapy

Current research efforts in both the basic and clinical sciences are improving clinicians' ability to more accurately target and treat GBM. In addition to targeted therapies, which will be discussed later in this paper, there have been advancements in the technological arena that are improving patient care.

### 3.1. Target Delineation

 MRI remains the gold standard for delineating tumor in both the pre- and postoperative setting. Gross tumor volume is felt to be best represented by areas of contrast-enhanced T1 signal while areas of T2/FLAIR enhancement reflect regions of infiltrative tumor. These volumes form the basis for radiotherapy target delineation. A limitation of contrast-enhanced MRI is that it relies on surrogate markers of tumor presence (tumor-associated breakdown of the blood-brain barrier and cerebral edema) versus a direct measure of actual gross tumor mass and spread. Functional imaging techniques such as positron emission tomography (PET) play a pivotal role in the staging and planning of cancers in other parts of the body. Unfortunately, the most widely used radiotracer [^18^F]-fluorodeoxyglucose is relatively insensitive at delineating malignancy in the brain due to the high basal metabolic rate of normal brain tissue [14]. Other clinically available radiotracers such as L-methyl-^11^C-methionine (MET) and *O-*(2-[^18^F]fluoroethyl)-L-tyrosine(FET) have shown promise in localizing gliomas. Recent studies have shown MET PET can more accurately identify areas of active tumor versus traditional MRI [15, 16]. MET is actively taken up by gliomas but shows only low uptake in normal brain. In a study of 14 patients with high-grade glioma, MET PET was highly correlated with areas of endothelial proliferation and mitotic activity [17], a more direct marker of tumor activity that can be visualized using MRI. In a study of 26 patients with GBM, Lee et al. [15] showed that 5 of 26 patients had areas of MET PET positivity outside radiotherapy volumes defined by MRI. All of these patients had noncentral failures. In 14 patients where MET PET-positive areas were covered in the high-dose radiotherapy volume, none had noncentral failures. FET PET has also been shown to correlate with areas of active tumor. In a study of 31 patients with GBM, FET PET was highly correlated with areas of active tumor on biopsy [18]. FET shows minimal uptake in macrophages or inflammatory tissues, indicating that it may be superior to MET in identifying areas of active tumor, especially in the postoperative setting. In a study comparing FET PET to MRI, Piroth et al. showed that areas active on FET PET not covered by MRI-based radiotherapy target volumes predict a shorter disease-free survival (5.1 versus 9.6 months) and overall survival (6.9 versus 20 months) further supporting the integration of metabolically based treatment planning methods into radiation planning for GBM [19]. An emerging technique that may also play an important role in target delineation for GBM is diffusion tensor imaging (DTI). DTI uses specialized MRI sequences to measure the diffusion of water in the brain, the greatest values of which lie along white matter tracts. Postmortem studies in GBM patients show that glioma cells tend to migrate the greatest distance from the primary site along these tracts. In a study of 14 patients with recurrent GBM, Krishnan et al. were able to show a strong relationship between the sites of recurrence and the DTI maps emanating from the primary site [20]. While none of the aforementioned imaging techniques have been tested in a phase III study, they provide promising tools by which clinicians cannot only more accurately identify areas of active tumor but also potentially predict their most likely route of spread to help refine radiotherapy treatment fields.

### 3.2. Disease Monitoring

Treatment-related effects such as postoperative scarring and hemorrhage, peritumoral edema, inflammation, and microvascular changes make radiographic assessment problematic as these changes can mimic progressive disease. This has been termed pseudoprogression. Additionally, given the high doses and often large volumes required to treat GBM, up to 30% of patients develop radionecrosis which can also mimic disease recurrence on MRI [21]. The front-line treatment for radionecrosis is steroids and time. Some patients require surgical resection for more advanced cases resistant to steroid treatment. The ability to diagnose true progression and initiate second-line therapy is paramount in a patient population with such poor disease-free and overall survival. Both PET imaging and magnetic resonance spectroscopy (MRS) have been shown to be superior to MRI in helping distinguish active disease from pseudoprogression or radionecrosis. The specificity of FET PET and MET PET to detect recurrent disease is approximately 90% and 70%, respectively [19]. Areas active on FET PET show a high correlation with biopsy-proven areas of active disease [18], and regions of enhancing tissue on MRI are often negative on FET PET. Numerous studies of magnetic resonance spectroscopy (MRS), which measures the differential concentrations of metabolites in the brain, also show improved accuracy in distinguishing recurrent disease versus MRI (71% versus 55%) [22]. Classic MRS is hampered by poor spatial resolution and nonvolumetric data; however, recent advances in technology now permit volumetric MRS which may improve its diagnostic value. Integration of these metabolically based imaging techniques may help to improve our ability to detect disease progression early and optimize 2nd-line therapy for patients.

### 3.3. Treatment in the Recurrent Setting

The vast majority of patients with GBM recur within 8–12 months following completion of therapy highlighting the importance of developing efficacious treatments for patients with recurrent disease. Studies examining reirradiation with or without chemotherapy have shown the most promising results. Given the fact that the majority of patients fail within 2 cm of the primary site, numerous studies examining the efficacy of stereotactic radiosurgery (SRS), as delivered by a variety of different methods, have been completed [23–25]. SRS provides a modest improvement in outcome but with significant risk of radionecrosis requiring surgical resection. Patients undergoing fractionated stereotactic radiotherapy (FSRT) at the time of recurrence have median survival ranging from approximately 7 to 14 months for all patients but with reduced rates of morbidity [23, 26–28]. Dosing regimens with a cumulative dose exceeding 40 Gy appear to be associated with a greater degree of radiation damage [29]. Response on postprocedure MRI seems to be an important prognostic factor for either SRS or FSRT. In a study of 36 patients (26 SRS, 10 FSRT) patients showing response on posttreatment MRI had median survival of 15.8 months versus 7.3 months for nonresponders [23].

Combination chemoradiotherapy has shown some efficacy in the recurrent setting. In a study of 25 patients with recurrent high-grade glioma (20 GBM, 4 anaplastic astrocytoma), treatment with FSRT (30 Gy in 5 fractions) plus concurrent bevacizumab resulted in posttreatment median survival of 12.5 months [30] with comparable toxicity rates as reported by other studies of GBM patients treated with bevacizumab. Three of 25 patients had to discontinue treatment due to CNS toxicity, wound complications, and bowel perforation. A recent phase I dose-escalation trial of gefitinib, an epidermal growth factor receptor (EGFR) tyrosine kinase inhibitor (TKI), plus FSRT (18 to 36 Gy in 3 fractions) in 15 patients (11 GBM, 4 anaplastic astrocytoma) showed this combination was well tolerated at all dose levels. Median progression-free survival was 7 months with a 6-month progression-free survival of 63% and a 1-year overall survival of 40%. Of course, given the mixture of grade III and grade IV histologies, it is difficult to compare outcomes with studies of GBM patients alone, but these initial results show promise. Studies combining cytotoxic chemotherapy with FSRT agents have shown similar response rates with acceptable toxicity profiles [31–33].

Recurrent malignant gliomas can also be managed with chemotherapy. Modifications in the dosing regimen of TMZ show modest improvements in progression-free survival. Standard dosing regimens of TMZ (200 mg/m^2^) produce a 6 month progression-free survival of 21% [34]. TMZ delivered in a low-dose, protracted schedule (75 mg/m^2^ for 21 days of a 28-day cycle) in an attempt to deplete MGMT produced PFS-6 of 30% [35].

## 4. Targeted Therapy

Considering glioblastoma treatment is the most expensive cancer treatment per capita in the United States, and outcomes are still so universally poor, there is a great need for more effective therapeutic options. Targeted therapy and personalized medicine are currently two of the more aggressively pursued ideas in cancer treatment. While targeted therapies aim to affect a specific alteration, most chemotherapies are generic, broad-based DNA-damaging agents that affect all cells in a similar manner. Targeted therapies offer the possibility of selectively killing cancer cells and sparing normal tissue. An example of a commonly targeted pathway in glioblastoma is the EGFR receptor tyrosine kinase.

## 5. Receptor Tyrosine Kinase (RTK) Inhibitors

### 5.1. EGFR

**Figure 1:**
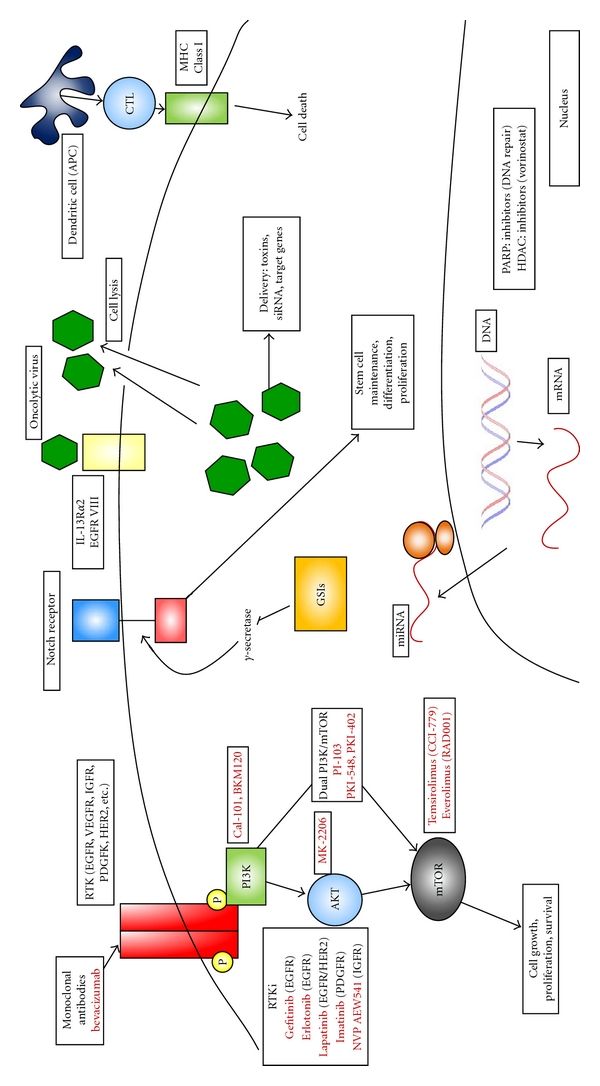
Novel therapies in GBM. RTKs and survival signaling pathways are major drug targets in GBM. Receptors have been targeted extracellularly by monoclonal antibodies or intracellularly at the tyrosine kinase domain. Major nodes in survival signaling pathways (P13K, AKT, mTOR) have been the focus of intense study and drug development. More recent approaches include stem-cell targeting (GSIs), inhibition of DNA rapair (PARP inhibitors), and targeting a host of cellular pathways through microRNA manipulation. Novel tumor cell killing approaches are also being studied. Oncolytic virus therapy, either alone or in combination with targeted agent delivery and immunotherapy, are being employed to efficiently kill tumor cells while sparing normal tissue. RTK, receptor tyrosine kinase; EGFR, epidermal growth factor receptor; VEGFR, vascular endothelial growth factor receptor; IGFR, insulin-like growth factor receptor 1; PDGFR, platelet-derived growth factor receptor; mTor, mammalian target of rapamycin; GSI, gamma-secretase inhibitor; APC, antigen-presenting cell; CTL, cytotoxic T lymphocyte, MHC class I, major histocompatibility complex I; PARP, poly(ADP-ribose) polymerase; HDAC, histone deacetylase inhibitor.

As mentioned above, EGFR is known to be an important player in gliomagenesis and in the aggressive and therapeutic-resistant phenotype demonstrated by this tumor. In addition to its critical role in several survival signaling pathways, alterations in EGFR are some of the most common mutations found in GBM. EGFR is overexpressed in approximately 50% of tumors, and of those nearly half express the constitutively active EGFRvIII mutant [36]. These combined facts have made this growth factor receptor a very popular target for molecular therapies. Many clinical trials have examined the efficacy of EGFR inhibitors, and to date there is little evidence to support their use in a monotherapy setting. While gefitinib, a selective inhibitor of EGFRs tyrosine kinase domain (Figure 1), is approved for use in non-small-cell lung cancer, a recent phase II clinical trial of gefitinib in newly diagnosed GBM patients by the Mayo/North Central Cancer Treatment Group showed no significant improvement in either overall survival or progression-free survival [37]. Another recent phase II trial studying the efficacy of erlotinib, which also inhibits the tyrosine kinase domain of EGFR (Figure 1), and TMZ with RT for newly diagnosed GBM had to be stopped short of full accrual due to the lack of benefit and the unreasonable toxicity, including at least three treatment-related deaths [38]. A different phase II trial of erlotinib in the same setting showed less toxicity and even demonstrated improved median survival (19.3 months versus 14.1 months) compared to historical controls [39]. Interestingly, the first study only escalated erlotinib dose to 150 mg/day while the second study, which showed improved toxicities as compared to historical controls, escalated to a maximum dose, in some patients, of 300 mg/day. Overall results from multiple clinical trials show monotherapy EGFR inhibition, or addition of these inhibitors to standard-of-care treatments has shown little benefit to patients and a dramatically increased toxicity profile.

In terms of current multitherapy strategies, combining several RTK inhibitors, or attacking several important glioma survival strategies, is likely to improve outcomes over monotherapies, while also possibly reducing toxicity. A recent study showed that EGFR expression in a GBM xenograft model increased the efficacy of an anti-vascular endothelial growth factor (VEGF) (vandetanib or cediranib) therapy in combination with irradiation when compared to xenografts lacking EGFR expression [40]. A preclinical study of monoclonal antibody inhibition of both EGFR and VEGFR-2 has demonstrated improved efficacy in an orthotopic xenograft model [41]. This research was not performed in the presence of IR and therefore may have less relevance to glioblastoma therapy. However, it does demonstrate the importance, as well as the complexity, of designing combination therapies. In a similar vein, it was recently shown that HER2 inhibition might help overcome EGFR resistance and increase radiosensitivity in a GBM cell model [42]. A different combination of EGFRvIII inhibition with C-met inhibition showed synergy against PTEN null/EGFRvIII positive tumors, a very aggressive tumor population [43]. With the promise shown, even novel inhibitors are being tested in combination. A recent study reported that inhibition of autophagy was able to enhance the cell-death-inducing capabilities of erlotinib in a glioma cell model [44]. Lovastatin, a member of the statin family (normally used to reduce cholesterol), was also shown to increase the efficacy of EGFR inhibitor therapies [45]. However, in a clinical phase I/II trial of lapatinib, a dual EGFR/HER2 inhibitor, showed poor results [46]. Even rational combinations targeting this pathway have not led to expected results, as shown by the underwhelming results in a recent phase II trial examining erlotinib therapy in combination with an mTOR inhibitor (sirolimus) [47]. While EGFR inhibitor combination treatments have produced improved results compared to monotherapy, we are still a long way from being able to determine what combinations will provide benefit for which patients.

### 5.2. Insulin-Like Growth Factor Receptor I (IGFR)

While EGFR has been the major focus of targeted receptor tyrosine kinase therapies, there has been work into other known survival signaling activating receptors such as IGFR. It has previously been established that there is significant cross-talk between IGFR and EGFR receptors, and the similar cellular responses to signaling through these receptors could play a large role in mediating resistance to anti-EGFR therapies [48]. In 2002, Chakravarti et al. showed upregulation of the IGFR gene in a GBM cell line resistant to AG1478, an EGFR TKI. In this work, they demonstrated that upregulation of IGFR in this resistant cell line correlated with sustained activation of the PI3K pathway. Cotargeting of the IGFR and the EGFR receptors led to increased apoptosis, as well as a reduction in invasive potential [49]. A more recent study demonstrated that combination of the IGFR inhibitor NVP-AEW541 with dasatinib led to increased apoptosis in GBM cell lines, but not in nonneoplastic human astrocytes, and synergistically inhibited clonogenic survival [50]. These studies highlight the possible efficacy of cotargeting IGF and EGF receptors to overcome therapeutic resistance and enhance therapeutic gain.

### 5.3. Platelet-Derived Growth Factor Receptor (PDGFR)

 PDGF signaling is another commonly altered signaling pathway in glioblastoma. A recent study in GBM cell lines showed that varying concentration of imatinib, a PDGFR inhibitor, had either cytostatic effects, at low concentrations, and possibly cytotoxic events at high concentrations [51]. Comparatively, another report in GBM cell lines showed treatment with imatinib actually led to the activation and sustained signaling through the ERK1/2, PI3K, and other important cell survival signaling pathways [52]. Some reports have even identified that PDGFR status was not predictive of imatinib efficacy even though it was shown to be a prognostic marker [53]. These results indicate that while PDGFR inhibition might be an interesting target, much more study is needed.

## 6. PI3K/AKT/mTOR Inhibitors

In addition to EGFR and other RTK therapies, there has been a major focus on inhibiting downstream survival signaling pathways stimulated by these receptors. The two most prominent and most studied pathways are the MAPK signaling cascade and the PI3K/AKT/mTOR pathway. Initially it was believed PI3K signaling was responsible for cell survival, while the MAPK pathway was involved in cell proliferation. Now, these two pathways are thought to share a significant amount of overlap and to both be involved in cell growth, proliferation, and survival. As targeted therapies have become a more important piece of the cancer treatment arsenal, these pathways have been the focus of a significant amount of research effort.

One of the initial works identifying PI3K inhibitors as viable for the treatment of GBM was a paper by Kubota et al. which showed the early PI3K inhibitor wortmannin sensitized GBM cells to radiation regardless of p53 status [54]. Wortmannin was then later shown to reverse the growth advantage seen in GBM cells which both lacked PTEN expression and overexpressed the EGFRvIII “always-on” variant growth receptor [55]. However wortmannin, while potent, has significant levels of nonspecific kinase inhibition and is soluble in organic solvents, which has limited its applicability for human clinical trials [56]. Following the proof of principle of this novel targeting therapy, many new PI3K inhibitors were developed and used in clinical trials. These include perifosine, cal101, px-866, pi-103, and others with some PI3K inhibitors even being specifically assessed in glioblastoma (XL765, XL147, and BKM 120) (Figure 1). Results of many of these trials have been poor; however, therapies using these drugs in combination with other inhibitors have recently become a focus.

In addition to PI3K inhibition, small-molecule inhibitors have been developed both upstream (i.e., AKT inhibitors) and downstream (i.e., mTOR inhibitors) in this pathway. AKT has been targeted because this kinase is the central node in the RTK/PI3K/AKT/mTOR signaling cascade. Direct inhibition of this molecule would prevent downstream signaling similar to RTKi or PI3K inhibition. The importance of developing these novel inhibitors at different stages of the signaling cascade has become even more obvious with the development of RTKi refractory tumors. Only one AKT inhibitor, mk-2206, has currently made it into phase II clinical trials. Recently GlaxoSmithKline has begun phase I trials with two different AKT inhibitors, with mixed results. The initial phase I study of drug GSK690693 was withdrawn, and trials of drug GSK2141795 are currently not recruiting patients. Also, a phase II trial of MK-2206 had been planned in recurrent glioma; however, that trial has since been withdrawn. However promising it has been preclinically, AKT inhibition has proven difficult to translate into clinical efficacy.

Probably the most targeted member of this pathway is the mammalian target of rapamycin (mTOR). In gliomas, it has been observed that the mTORC2 complex promotes growth and cell motility [57]. An early study demonstrating efficacy of targeting this pathway showed increased radiosensitization of a U87 xenograft [58]. Based on available data, it seems likely that in order to effectively block mTOR activity in cells, both mTORC1 and mTORC2 complexes will need to be targeted [59]. These preclinical results have been critical to planning the numerous clinical trials that have been performed with mTOR inhibitors in glioblastoma. A phase I trial of rapamycin in PTEN-deficient glioblastoma patients, while showing some promising results, also demonstrated the inherent difficulties of targeting this protein. In this trial, multiple patients were observed who showed elevated levels of pAKT following mTOR inhibition, which was correlated with shorter time to progression [60]. The AKT activation observed was likely due to alteration of signaling feedback loops, again highlighting the complexity of targeted therapy. Combination therapy to block these feedback loops may also improve efficacy [61]. Despite this complexity, promising results have pushed mTOR inhibitors to further trials. Several of these mTOR inhibitors have been or are currently being tested in the clinical trials setting specifically in gliomas, including temsirolimus, everolimus (RAD001), and sirolimus. Temsirolimus (CCI-779) has been the most extensively studied drug in clinical trials. A phase I study determined the clinically effective dose to be 250 mg IV weekly [57]. Phase II trials with CCI-779 as a monotherapy in recurrent GBM showed no effectiveness despite low toxicity and initial disease stabilization [62]; however, a North Central Cancer Treatment Group study showed a statistically significant time to progression increase in temsirolimus responders (5.4 months versus 1.9 months, 2.3 months overall) [63].

Because of the promise of combination therapy, currently there is a significant emphasis on dual PI3K/mTOR inhibitors. Several novel small-molecule inhibitors have been developed that have dual specificity for these targets. XL765 has recently been shown to reduce cell viability in vitro and in limited animal study showed a possible effectiveness when combined with TMZ therapy [64]. Similarly PKI-587 and PKI-402 were shown to have a strong in vitro antitumorigenic effect across multiple cell types including glioma cells, while also slowing tumor growth in xenograft models [65, 66]. Another dual PI3K/mTOR inhibitor, PI-103, which is known to have monotherapy efficacy in glioma [67] was recently shown to specifically reduce tumor volumes in combination with NSC-delivered s-trail in an orthotopic intracranial xenograft model [68]. PI-103 combination therapy has also proven effective in sensitizing cells to both chemotherapy [69] and radiation [70] through reducing DNA damage repair. RAD001 is currently being used in multiple combination treatment studies, including studies employing an oncolytic virus [71], Raf inhibitors [72], and VEGFR-2 [73]. In a GBM orthotopic xenograft model, it was shown however that PTEN does not serve as a predictive marker for RAD001 effectiveness, despite the importance of PI3K/AKT signaling activation in mTOR upregulation [74]. There has also been evidence for targeting this signaling pathway from both ends, with a report showing rapamycin promotes a response to EGFR inhibitors in either PTEN-sufficient or PTEN-deficient GBM cells by reducing tumor cell growth. This treatment combination also results in tumor cell death in PTEN-deficient cells [75]. While there is hope clinical trials with these novel dual-targeting agents will demonstrate better efficacy than current monotherapies, there are still significant difficulties in trying to determine the best role for these targeted therapies in cancer treatment.

## 7. Antiangiogenic Therapies

Antiangiogenic therapy, which has been well studied in many types of cancer, has also emerged as a novel therapy for glioblastoma. Glioblastoma is characterized by vascular proliferation or angiogenesis [76], and advances in molecular biology have allowed us to target angiogenesis of glioblastoma. VEGF, a critical mediator of angiogenesis, is highly expressed in glioblastoma and regulates tumor angiogenesis [77, 78]. Preclinical studies have shown that VEGF inhibitors inhibit the growth of glioma cells [79, 80]. Antiangiogenic therapies for glioblastoma are currently the most advanced of any targeted therapy, and many clinical trials have demonstrated their efficacy. In fact, bevacizumab has been approved by the US food and Drug Administration in the setting of recurrent glioblastoma.

Bevacizumab is a humanized monoclonal antibody against VEGF and prevents the activation of VEGF receptor tyrosine kinases (Figure 1) [81]. This drug is considered a well-established antiangiogenic therapy in several angiogenic tumors [82]. In glioblastoma, a phase II study of the addition of bevacizumab to the standard treatment of TMZ and radiotherapy was conducted for 70 newly diagnosed patients [83]. The median overall survival and PFS were 19.6 and 13.6 months, respectively. Another phase II study of additional bevacizumab to standard therapy showed median PFS was 13.8 months in 125 newly diagnosed glioblastoma patients [84]. Recently two clinical trials of bevacizumab have been reported in 2011 ASCO annual meeting for newly diagnosed glioblastoma. Vredenburgh et al. performed a phase II study of bevacizumab, TMZ, and radiotherapy followed by bevacizumab, TMZ, and oral topotecan [85]. The 6-month event-free survival was 90%, and median overall survival has not been reached. Although the regimen was tolerable, there were 2 treatment-related deaths, including CNS hemorrhage and pneumonitis. Omuro et al. conducted a phase II trial of bevacizumab, TMZ, and hypofractionated stereotactic radiotherapy for newly diagnosed glioblastoma patients with tumor volume under 60 cc [86]. The median PFS was 11 months, and objective response rate was 90%. Furthermore, 1-year overall survival was 90% with median follow-up of 13 months. Despite a more aggressive radiotherapy schedule, the regimen was well tolerated and had promising results. Additional bevacizumab seems to have favorable effects; however, it is still unclear whether this regimen can improve overall survival. Two randomized phase III trials, RTOG 0825 and AVAGLIO, are ongoing to demonstrate the efficacy and safety of combined therapy of bevacizumab, TMZ, and radiotherapy for newly diagnosed glioblastoma [87, 88]. The addition of bevacizumab to TMZ and radiotherapy is expected to become frontline treatment for glioblastoma, and is already thought of by some as nearly a part of standard of care.

While bevacizumab has been thoroughly investigated in clinical and preclinical studies, other drugs have also been studied as antiangiogenic therapy for glioblastoma patients [89]. Cilengitide, which is selective for *α*v*β*3 and *α*v*β*5 integrin receptors, is considered a novel antiangiogenic therapy for glioblastoma. A phase II study of recurrent glioblastoma treated by cilengitide showed that 6 month PFS was 15%, and treatment was well tolerated [90]. Furthermore a phase I/IIa study of cilengitide combined with TMZ and radiotherapy was performed for 52 newly diagnosed glioblastoma patients [91]. This regimen was well tolerated and showed promising results with median overall survival of 16.1 months. Currently, two randomized trials, CENTRIC and CORE, are ongoing and are expected to show the benefits of additional cilengitide to standard therapy for newly diagnosed glioblastoma patients [88, 92]. Other antiangiogenic therapies (e.g., VEGF receptor tyrosine kinase inhibitors) have also been performed in clinical and preclinical studies [89], although there have not been any drugs to show strong antiglioma effects compared with bevacizumab. Further investigation is warranted to establish the efficacy and safety of novel antiangiogenic therapy for glioblastoma.

## 8. Novel Targeted Therapies

### 8.1. Notch Inhibitors

One of the most controversial topics in cancer biology is the theory of cancer stem cells or tumor-initiating cells. Despite the split opinions regarding the existence of this cell population, mounting evidence has spurred development of novel therapeutics to target this proposed group of cells. While proper definition and identification of this stem cell population is still ongoing, researchers have used clues about pathways critical to known stem cell populations to design novel therapies. One such pathway is the notch signaling pathway, which is important in both normal and neoplastic development in the central nervous system by controlling proliferation, apoptosis, stem cell maintenance, differentiation, and homeostasis [93]. Specifically in gliomas, notch has been linked to overexpression of EGFR [94]; however, it is more likely notch's role in the maintenance of stem cell populations that make it an interesting therapeutic target [95, 96]. Gamma secretase inhibitors (GSIs), which are known to inhibit notch, have been shown to inhibit glioma stem cell growth [97], while overexpression of notch induces tumor growth and can be blocked by treatment with GSIs (Figure 1). While notch inhibition is still very novel, it has shown efficacy in preclinical models and shows a strong possibility for combination therapy targeting both tumor cell bulk with conventional therapies as well as the tumor-initiating cell population.

### 8.2. Virotherapy/Gene Therapy for GBMs

Cancer therapy using viruses comes in a variety of approaches including direct viral cytotoxicity and targeted toxin delivery. The use of viruses to deliver tumor suppressors, or siRNAs, to knockdown oncogene expression, immune modulating compounds, or antiangiogenic compounds, is also underway, yet these attempts are often designed in combination, or as a method to enhance oncolytic viruses (Figure 1).

The general goal behind direct viral cytotoxicity or oncolytic virotherapy is to design a virus that specifically and faithfully infects and replicates only in tumor cells. This is usually accomplished by attenuating the virus to restrict their replication to actively dividing cells (i.e., tumor cells) while sparing normal, nonreplicating tissue. Viruses such as herpes simplex virus 1 (HSV), adenovirus, and reovirus have been attenuated so as to conditionally replicate within cancer cells [98–100]. Oncolytic viral therapy has undergone major changes, using the virus not only as a cytotoxic therapy, but also as a delivery mechanism. Researchers have used oncolytic HSV-1 to deliver vasculostatin, an antiangiogenic compound [101], as well as chondroitinase ABC I [102]. This method has demonstrated significantly enhanced therapeutic efficacy over virus alone. Similar approaches with adenoassociated virus (aav) particles [103] have shown codelivery of oncolytic virus with antitumor molecules has enhanced efficacy and improved survival in xenograft models. Each of these oncolytic viruses has also been examined in phase I clinical trials in glioma and have been shown to be well tolerated [104–106].

Targeted toxin delivery is similar to oncolytic virus therapy in that it is a virus-mediated cytotoxic therapy. While oncolytic viruses are directly responsible for tumor cell death, the strategy for this therapy is to use nonreplicating virus particles, such as aav, to deliver a powerful toxin, that is, *Pseudomonas* exotoxin (PE), specifically to cancer cells by targeting preferentially expressed receptors. Some of the receptors include the IL-13R variant *α*2, which varies from the receptor expressed on normal brain cells [107], as well as EGFR [108] or the EGFRvIII variant [109]. Clinical trials have been performed with an IL-13R*α*2 targeting virus delivering cintredekin besudotox (CB). A phase III study of this therapy compared to Gliadel Wafer administration at first recurrence showed no survival benefit [110]. In general, despite promising preclinical results with viral therapy for gliomas, clinical trials resulting from this work have yet to show any significant survival benefits. This pattern is not uncommon in cancer therapy, yet it does point out the need for more research. Despite the poor clinical trial results to date, the positive data coming from viral therapy research indicate that this form of therapy has significant future potential.

### 8.3. Immunotherapy

One alternative to target gliomas without affecting normal cells is by utilizing the body's natural defenses to kill tumor cells. There are currently two main immunotherapy strategies being tested against gliomas: adoptive immunotherapy and active immunotherapy.

Adoptive immunotherapy is the process of stimulating immune cells *ex vivo* and then readministering them to the patient in hope of therapeutic benefit. This can be done either intravenously or directly into the tumor. The two major cell types used are lymphocyte activated killer (LAK) cells or cytotoxic T lymphocytes (CTLs). Recent reports have identified a synergistic response between cytokine-induced killer cells (CIKs) and TMZ [111]. Specific targeting of HER-2 by T cells was shown to induce regression in HER-2-positive autologous tumor xenografts as well as to target the tumor-initiating cell (TIC) population, demonstrating a targeted approach may prove to be more effective [111, 112]. A significant number of phase I or phase I/II trials have been performed using LAK cells or CTLs. These trials were largely in the recurrent setting and demonstrated limited efficacy combined with significant rates of toxicity for LAK cells [113], and better tolerance and improved survival for CTLs [113]. While responses to the adoptive therapies have shown only limited efficacy to date, it is possible that combination therapy or more targeted immunotherapeutic approaches may be of use.

Active immunotherapy is similar to vaccination, the idea being to stimulate the patient's immune system by using tumor-related sources of antigen (whole tumor cells, tumor protein lysates, mRNA, synthetic peptides). These sources of antigen can either be injected alone or coupled to dendritic cells [113]. Dendritic cells are powerful antigen-presenting cells, and dendritic cell therapy is designed to increase antigen presentation by incubating tumor antigens with these cells before injecting them back into the body (Figure 1). This method of active tumor immunotherapy has been the most extensively studied with widely varying results. Some of the trials performed using this method have shown promising results, while others demonstrated no benefit [113]. A major problem in interpreting results from these trials is the wide variation in protocols for everything from acquiring cells, type of tumor antigen chosen, and number of cells used. However, targeting this type of therapy may lead to enhanced clinical benefit. One major trial using patient tumor cell cultures infected with Newcastle Disease virus followed by gamma irradiation demonstrated significant increases in progression-free survival, 40 weeks versus 26 weeks, as well as overall survival, 100 weeks versus 49 weeks [114]. This trial also reported increased 1-year (91% versus 45%), 2-year (39% versus 11%), and long-term survivors (4% versus 0%). These results are very promising; however, the trial was nonrandomized and studied a limited number of subjects (23 patients receiving immunotherapy with 87 controls). Controls were also not treated using current standard of care as this trial was performed in 2004 before the results of the Stupp trial were published. Human cytomegalovirus (CMV) has been identified to be associated with tumors in a significant proportion of glioblastoma patients (50–90%) [115, 116]. Currently two clinical trials are ongoing at the Duke Brain Tumor Immunotherapy Program attempting to utilize this knowledge. The Vaccine Therapy in Treating Patients with Newly Diagnosed Glioblastoma Multiforme (ATTAC) [117] and the Evaluation of Recovery From Drug-Induced Lymphopenia Using Cytomegalovirus-Specific T-Cell Adoptive Transfer (ERaDICATe) [118] trials are either recruiting or in a data analysis phase, with results likely to be reported soon.

### 8.4. DNA Damage

DNA damage repair is a double-edged-sword in the cancer world. Lack of proper DNA repair can lead to genomic instability and the generation of cancer. However, once cancer is established, DNA repair genes undermine many effective cancer therapeutics. Both radiotherapy and chemotherapies are designed around a DNA-damaging strategy designed to induce cell death and tumor regression. In the presence of DNA repair proteins, these therapies have reduced efficacy. By targeting DNA repair proteins in cancer cells, we can again render them sensitive to radiation and chemotherapies. PARP plays a role in single stranded DNA, repair and inhibitors are currently being tested in a number of cancer sites. If PARP is inhibited, single-stranded nicks will not be repaired and will lead to DNA strand breaks during replication. Double-stranded DNA breaks are extremely toxic lesions to cells, and thereby PARP inhibition should increase cell death in proliferating cells (Figure 1). PARP inhibitors E7016 and AZD2281 have been shown to radiosensitize GBM cells both *in vitro *and* in vivo* [119, 120], with an enhanced effect when combined with heat shock protein 90 (HSP90) inhibition [121]. PARP inhibitors have also been shown to increase the efficacy of chemotherapies such as DNA topoisomerase I poisons, TMZ, iriniotecan, or cilengitide [122–124]. It has also been observed that PTEN loss can negatively affect homologous recombination, thereby increasing the efficacy of PARP inhibition as well as other DNA-damaging modalities [125]. In addition to PARP inhibition, other targets have been identified to utilize DNA repair as a therapeutic strategy. Inhibition of PP2A has been shown to augment DNA-damaging agents by inducing Plk-1 and AKT activation and decreasing p53 expression which has led to complete remission or significant tumor regression, when combined with TMZ or doxorubicin, in a large number of tumors in a xenograft model [126].

### 8.5. Autophagy

Autophagy is an evolutionarily conserved process through which the cell is able to degrade damaged organelles and other cell components [127]. Reports identify autophagy to serve a dual role in cancer. Limited and controlled autophagy can be a survival method for cancer cells which allows them to overcome current therapies such as chemotherapy and radiation [128–130]. However, autophagy has also been identified as a possible therapeutic target because sustained and uncontrolled autophagy can lead to cell death [131, 132]. This process is interlinked with several crucial cancer survival pathways including p53 signaling and the PI3K pathway, as well as apoptotic signaling molecules like Bif-1 [127]. Currently, attempts are being made to control this switch and tip cells into a pro-death state. This work is similar to the rationale behind apoptotic research. Resistance to apoptosis is one of the major hallmarks of many cancer types, and the ability to regulate this cellular process would allow for not only increased efficacy of current treatments but also a novel target for future therapy. As with most therapies discussed so far, combination therapy will likely play a key role in future treatment plans. Recent studies have demonstrated autophagy can be induced in glioma cells by current standard-of-care therapy [130, 133] and AKT signaling plays a major role in the prosurvival effects of this process [129, 134]. This data indicates that cotargeting AKT will be important in regulating and controlling the prodeath role of autophagy in glioma.

### 8.6. HDAC Inhibitors

HDAC inhibitors have been studied in the setting of GBM and shown positive indications in both preclinical and clinical testing. In preclinical models, HDACs have been shown to sensitize cells to chemotherapy [135], to have antiproliferative activity by increasing PTEN and AKT expression while reducing phosphorylation of the proteins to their active forms [136], to increase apoptosis in GBM cells through activation of the JNK pathway and reduction in telomerase activity [137], and to sensitize cells to radiation [138]. In addition, in a phase II clinical trial, the potent HDAC inhibitor, vorinostat, was shown to have modest single-agent efficacy and to be well tolerated [139].

### 8.7. MicroRNA

As microRNAs have become better studied, the possible roles in therapeutic scenarios have increased. Issues still remain in targeted delivery of these microRNA constructs to tumor cells, but many possible targets have been identified for almost all cancer types. In glioblastoma, some of these targets include miR-124 and miR-137, which could target both tumor-initiating cells, by inducing differentiation, and normal glioblastoma cells by arresting cell growth [140]. The miR 302–367 cluster has also been shown to induce TIC differentiation as well as to reduce the infiltrative properties of these cells [141]. A miR-21 inhibitor has been shown to sensitize the glioblastoma cell line U251 to ionizing radiation [142]. miR-34-a has been shown to inhibit glioblastoma growth by targeting the c-Met and notch pathways, two well-known signaling pathways linked to glioma pathogenesis [143]. miR-10b has been linked to glioblastoma cell growth [144], and miR-124a has been linked to migration and invasion [145]. This sampling of microRNA targets linked to glioblastoma growth, survival, TIC maintenance, and migration/invasion underscores the possible therapeutic application of microRNAs to regulate major pathways linked to disease severity.

## 9. Discussion

In this paper we have discussed RTK inhibition, angiogenesis inhibitors, and the PI3K/Akt/mTOR inhibition in detail due to the substantial amount of research conducted in these areas and have also briefly discussed several very novel areas of research including Notch inhibition, viral and immunotherapies, and DNA repair pathways. Within the heavily researched pathways, the overarching theme in glioblastoma therapy is that monotherapies demonstrate limited efficacy. Because single-agent therapies have shown no significant benefit, it is critical to begin designing rational combinations. Different RTK inhibitors combined with PI3K/mTOR dual inhibitors or antiangiogenic agents combined with Akt inhibition are already being examined. It is likely that many of the novel therapies discussed in this work will demonstrate greater efficacy when paired with the more studied targeted therapies. Because many of these targets are within the same signaling cascade, inhibiting pathways horizontally rather than vertically should remove some of the compensatory mechanisms glioblastomas use to overcome treatment. It is also important to note that many of the molecular biology advancements will be augmented by advancements in current treatments. Improved tumor border delineation or detection of microscopic disease will enhance the efficacy of upfront surgical and radiotherapy interventions while better methods for posttreatment image surveillance will improve treatments in the recurrent setting. Critically, it should be acknowledged that these therapies will need to work in conjunction with the current standard of care, highlighting treatments that can serve as radio-or chemosensitizers. Glioblastoma carries a very poor prognosis, but with improved technology and novel, personalized, rational, targeted therapies patient survival and quality of life will be greatly improved.
